# Long-term results of radical pericardiectomy for constrictive pericarditis in Korean population

**DOI:** 10.1186/s13019-019-0845-7

**Published:** 2019-02-06

**Authors:** Min Suk Choi, Dong Seop Jeong, Jae K. Oh, Sung-A Chang, Sung-Ji Park, Suryeun Chung

**Affiliations:** 1 Department of Thoracic and Cardiovascular Surgery, Samsung Medical Center, Sungkyunkwan University School of Medicine, Seoul, Republic of Korea; 2 Department of Thoracic and Cardiovascular Surgery, Dongguk University Ilsan Hospital, Dongguk University School of Medicine, Goyang, Republic of Korea; 3 Department of Cardiology, Department of Medicine, Samsung Medical Center, Sungkyunkwan University School of Medicine, Seoul, Republic of Korea; 4 Division of Cardiovascular Diseases and Internal Medicine, Mayo Clinic, Rochester, MN USA

**Keywords:** Constrictive pericarditis, Pericardiectomy

## Abstract

**Background:**

The extent of pericardiectomy is an important issue in constrictive pericarditis but its impact on long-term outcomes has been rarely reported. We compared long-term results of radical pericardiectomy with conventional phrenic to phrenic pericardiectomy.

**Methods:**

Ninety patients who underwent pericardiectomies between February 1995 and April 2015 were reviewed retrospectively. They were classified into conventional (*n* = 37) and radical (*n* = 53) groups according to pericardiectomy being performed anterior or posterior to the phrenic nerves, respectively. The follow-up duration at outpatient clinic was 37.6 (11.7, 86.6) months and the survival data until 91.6 (54.5, 147.0) months were obtained. The last echocardiographies were done at 22.4 (4.35, 60.85) months.

**Results:**

The early mortality rate was 4.4% (4/90). They all belonged to the conventional group and died of low cardiac output syndrome. The survival rate was higher in the radical group (*P* = .032, 74.7 ± 9.2% versus 50.4 ± 11.9% in 20 years). NYHA class of both groups had recovered until the last follow-up but the radical group showed better recovery (*P* < .001). The conventional pericardiectomy (HR = 6.181; 95% CI (1.042, 36.656)), redosternotomy (HR = 6.441; 95% CI (1.224, 33.889) and preoperative grade of tricuspid regurgitation (HR = 15.003; 95% CI (1.099, 204.894) were associated with late mortality. Right ventricular systolic pressure decreased, and pericardial thickening resolved only in the radical group with significant intergroup differences as time went on. Tricuspid regurgitation worsened after the operation in both groups, but it deteriorated more in the conventional group. However, it improved over time in the radical group.

**Conclusions:**

Radical pericardiectomy led to greater improvement in right ventricular systolic pressure and lesser deterioration of tricuspid regurgitation with the passage of time than did the conventional procedure. Conventional pericardiectomy and preoperative higher grade tricuspid regurgitation were associated with long-term mortality.

**Electronic supplementary material:**

The online version of this article (10.1186/s13019-019-0845-7) contains supplementary material, which is available to authorized users.

## Background

Constrictive pericarditis has a thickened, inflamed, adherent, or calcified pericardium that limits diastolic ventricular filling of the heart [1]. Pericardiectomy restores them and postoperative clinical outcomes and prognostic factors have been reported [2–9].

**Fig. 1:**
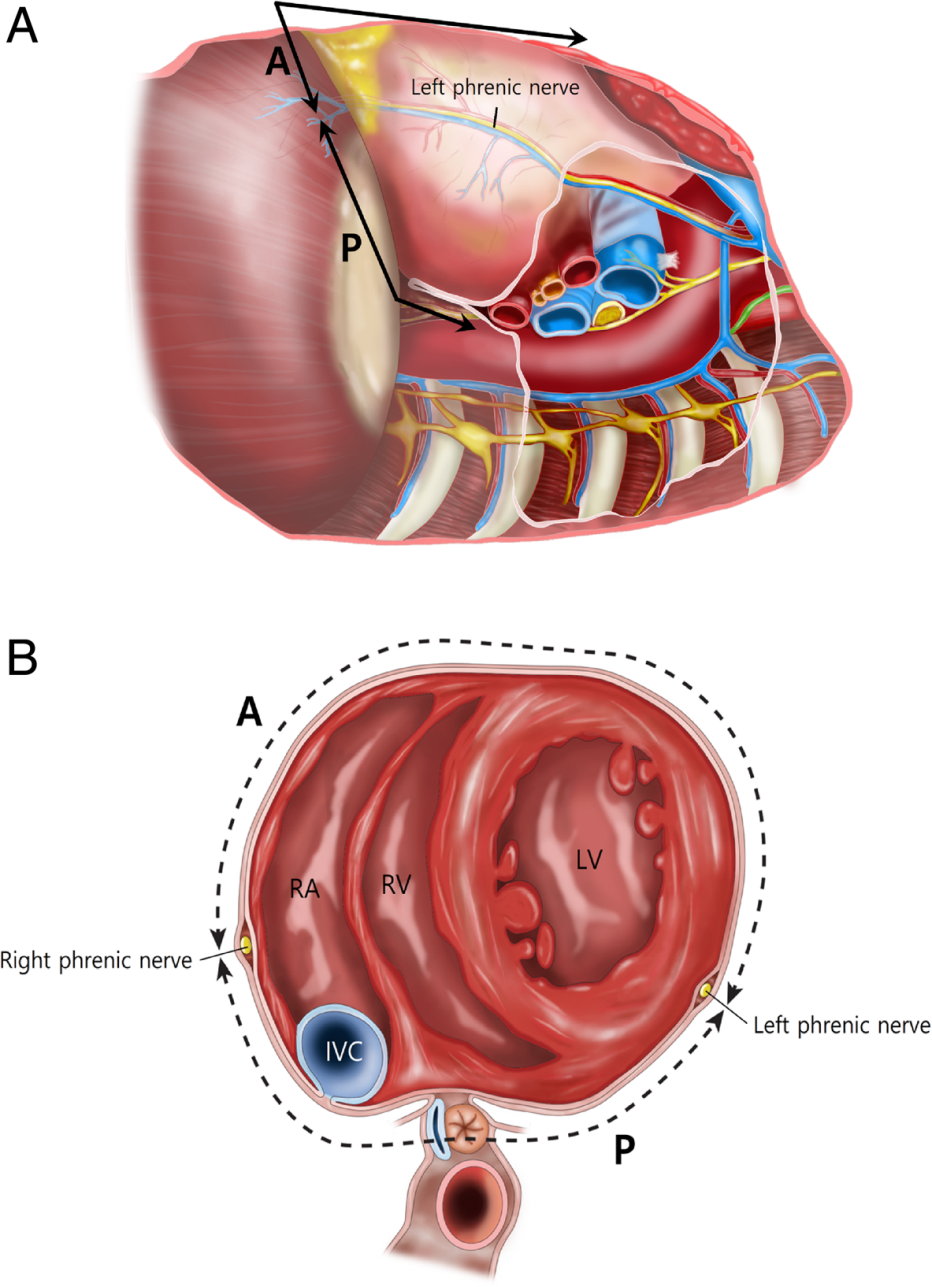
Left lateral (**a**) and axial view (**b**) of the heart. The range of conventional pericardiectomy was A and that of radical pericardiectomy was A and P. (A, pericardium anterior to phrenic nerve; IVC, inferior vena cava; LV, left ventricle; P, pericardium posterior to phrenic nerve; RA, right ventricle; RV, right ventricle)

A wider surface than we think exists posterior to the phrenic nerve (Fig. 1) [10]. However, pericardiectomy posterior to the phrenic nerve is not easy because it could make vital signs unstable if a heart is bent anteriorly. Therefore, it is reasonable to assume the extent of pericardiectomy would make better outcomes. Many papers have suggested the prognosis would improve as more pericardium is resected, but only Chowdhury et al. [3] have shown this by comparative analyses according to the extent of pericardiectomy.

## Methods

### The aim

To confirm the merit of more aggressive pericardiectomy, we compared long-term results of more extensive pericardiectomy with conventional phrenic to phrenic pericardiectomy.

### Patients

Ninety patients who underwent pericardiectomies between February, 1995 and April, 2015 were reviewed retrospectively. The most common etiology was idiopathic, followed by tuberculosis and previous chest surgery (Table 1). The condition was diagnosed almost all by echocardiography, but cardiac catheterization (30%), computed tomography (96%), and magnetic resonance imaging (14%) were also performed. Operative indication was congestive heart failure caused by constrictive pericarditis. Patients were divided into conventional and radical groups according to the extent of pericardiectomy. Baseline characteristics were not different between groups, except for central venous pressure (CVP) and pericardial thickening (Table 2). Our Institutional Review Board approved this study and waived the requirement for individual consent.

**Table 1:** Disease Etiology

Causes	Conventional group	Radical group	N (%)
Idiopathic cause	9	25	34 (37.8%)
Tuberculosis	12	17	29 (32.2%)
Previous chest surgery	4	7	11 (12.2%)
Post-radiation	4	1	5 (5.6%)
Uremic disease	1	2	3 (3.3%)
Metastatic carcinoma	3	0	3 (3.3%)
Autoimmune disease	2	0	2 (2.2%)
Prior chest trauma	0	1	1 (1.1%)
Post-infection	1	0	1 (1.1%)
Multifactorial cause	1	0	1 (1.1%)

**Table 2:** Baseline Data

Profile	Conventional group	Radical group	*P* value
Number of patients, n	37	53	
Age, years	52.4 ± 17.2	53.7 ± 13.5	.976^a^
Male: Female, n	26: 11	39: 14	.730^b^
Hypertension, n (%)	7 (18.9%)	9 (17.0%)	.813^b^
Diabetes mellitus, n (%)	5 (13.5%)	7 (13.2%)	1.000^c^
Chronic renal failure, n (%)	3 (8.1%)	2 (3.8%)	.398^c^
Cerebrovascular accident, n (%)	3 (8.1%)	1 (1.9%)	.302^c^
Atrial fibrillation, n (%)	12 (32.4%)	19 (35.8%)	.737^b^
Liver cirrhosis, n (%)	13 (35.1%)	15 (28.3%)	.491^b^
Ascites, n (%)	20 (54.1%)	28 (52.8%)	.909^b^
Previous open heart surgery, n (%)	4 (10.8%)	6 (11.3%)	1.000^c^
NYHA class I ~ II: III ~ IV, n	23: 14	40: 13	.175^b^
Serum hemoglobin, g/dL	11.4 (10.2, 13.0)	13.9 (11.9, 14.9)	.052^d^
Serum sodium, mEq/L	137.0 (134.0, 139.0)	138.6 (137.0, 140.0)	.019^d^
Serum albumin, g/dL	3.7 ± .6	3.9 ± .5	.253^a^
Serum bilirubin, mg/dL	0.9 (0.6, 1.4)	1.3 (0.8, 1.6)	.885^*d^
Serum creatinine, mg/dL	1.1 (0.8, 1.4)	1.0 (0.8, 1.1)	.539^d^
MELD score	12 (8, 16)	11 (9, 13)	.318^d^
Serum NT-proBNP, pg/ml	1324.0 (343.5, 1690.0)	582.1 (275.6, 688.9)	.066^d^
Preoperative CVP, mmHg	18.5 ± 6.1	16.2 ± 4.8	.032^a^
Pericardial thickening, n (%)	29 (80.6%)	49 (96.1%)	< .001^b^
EuroSCORE	3.0 (2.0, 6.0)	3.0 (2.0, 4.0)	.527^d^
Logistic EuroSCORE	2.3 (1.5, 4.3)	2.1 (1.5, 3.5)	.970^d^

^a^Student’s t-test; ^b^Chi-squared test; ^c^Fisher’s exact test; ^d^Mann-Whitney test; CVP, central venous pressure; MELD, model for end-stage liver disease; NT-proBNP, N-terminal prohormone of brain natriuretic peptide; NYHA, New York Heart Association

### Operation

The operations were performed by ten surgeons. Conventional pericardiectomy removed the anterior and diaphragmatic pericardium anterior to the phrenic nerves. Radical pericardiectomy additionally removed the pericardium posterior to the phrenic nerves to see the coronary sinus or pulmonary veins. Patients were included in the conventional group if the pericardium posterior to the phrenic nerves was not removed, even though the diaphragmatic pericardium was removed (Fig. 1).

**Fig. 2:**
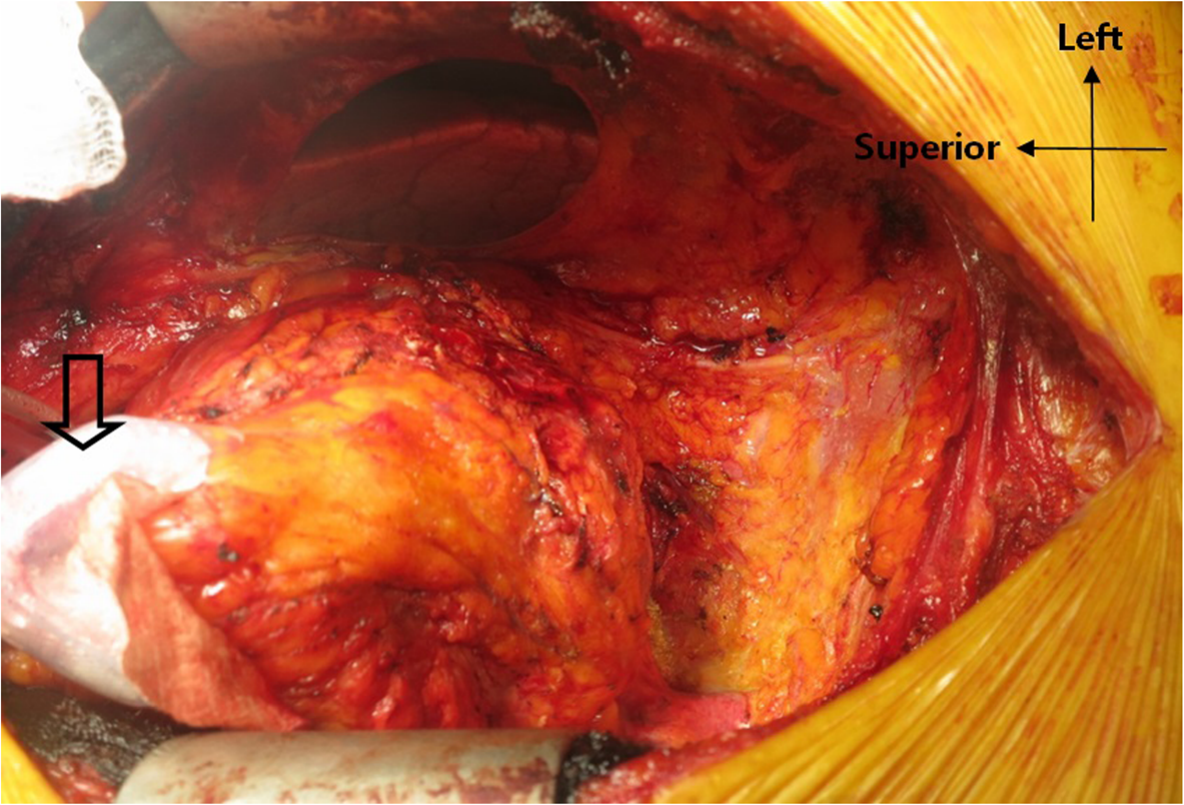
Radical pericardiectomy resects the pericardium until the inferior vena cava, coronary sinus and pulmonary veins are seen. A cardiac positioner (arrow) is useful to retract the heart

The surgical sequence of the radial pericardiectomy is as follows. Median sternotomy is performed after general anesthesia. If cardiopulmonary bypass (CBP) is being planned, then initial dissection is performed over the aorta. Dissection around the right atrial auricle is performed for venous cannulation. After cannulation, normothrermic bypass is initiated and the dissection proceeds. The anterior pericardium is initially divided in the midline or wherever a nonadhesive pericardial space is present. The dissection of the pericardium off the heart is usually done with metzenbaum scissors or electrocautery. A cardiac positioner which is used during off-pump coronary bypass grafting is useful to retract the heart (Fig. 2) [11]. The dissection proceeds laterally on the right side to remove the pericardium surrounding the right atrium until it extends to about 1 cm anterior to the right phrenic nerve. The dissection proceeds to the left side until it ends approximately 1 cm anterior to the left phrenic nerve. The anterior pericardium is then removed. Dissection proceeds between the pericardium and the posteroinferior ventricular wall until the inferior vena cava, coronary sinus and pulmonary veins are seen and then the diaphragmatic and posterior pericardia are resected (Fig. 2). If CPB is not being planned, the initial pericardiectomy is done around the left heart because the pericardiectomy around the right heart without full release of the left heart can cause right heart failure.

Which surgical technique would be chosen was determined during the operation rather than preoperatively or according to the surgeon’s preference. Conventional pericardiectomy was done if the posterior pericardial constriction was little or the pericardiectomy was difficult due to dense adhesion extending into the myocardium or hemodynamic instability. The number of each operative technique which was performed every year were described in Additional file 1.

All patients except one (via left thoracotomy), were approached via median sternotomy, among which 11 (12%) cases were redo-sternotomies. CPB was done in 75 (83%) patients and was usually used when radical pericardiectomy was done, epicardial constriction was extensive, a calcific plaque invaded the myocardium, or another cardiac operation was planned. Fifteen out of those 75 patients needed aortic cross-clamping for concomitant cardiac operations.

### Clinical outcomes

Medical records were reviewed and the recent survival data were acquired from the national medical insurance registry. Early mortality meant in-hospital mortality and late mortality was mortality after discharge. New York Heart Association (NYHA) class which reflects heart failure was graded. The follow-up duration was 37.6 (11.7, 86.6) months and the survival data until 91.6 (54.5, 147.0) months were obtained.

M-mode, two-dimensional, and pulsed-wave Doppler echocardiography were performed. Tissue Doppler echocardiography has been done gradually since 2000 and routinely since 2005. Doppler echocardiographs were reviewed by a certified echocardiographer who did not have any knowledge of the patients. Preoperative, immediate postoperative (0.3 (0.2, 0.4) month) and last (22.4 (4.35, 60.85) months) echocardiographic images were analyzed. The immediate postoperative echocardiography was done before the discharge while the last echocardiography meant the last echocardiography after the discharge.

### Statistics

IBM SPSS Statistics (version 19, SPSS Inc., Chicago, IL) was used. Data were presented as means, medians, or proportions. Means ± standard deviations were calculated when continuous variables were normally distributed. If the distribution of the data was skewed, the median was calculated, and the 25th and 75th percentile values were placed in parentheses. Categorical variables were compared with the Chi-squared test or Fisher’s exact test according to the expected frequencies. Continuous variables of two groups were compared by the two-sample t-test or Mann-Whitney test according to the normal distribution of the data. Continuous variables of three groups were compared by one-way ANOVA or the Kruskal-Wallis test according to the normal distribution and equal variance of the data. Then, the Scheffe test using ranks was performed for subgroup comparisons if a significant difference was found in the Kruskal-Wallis test. A correlation between ordinal variable and continuous variable was analyzed by Spearman correlation analysis. The Kaplan-Meier method estimated survival rates and the intergroup difference was assessed by log-rank test. To identify factors associated with survival, variables were checked initially by log-rank test for categorical variables or Cox proportional hazard model for continuous variables (univariate analysis, Additional file 2). Variables with a *P* value less than .20 in univariate analyses or interesting variables (Additional file 2) were then tested in the Cox proportional hazard model to identify factors associated with survival (multivariate analysis). A *P* value less than .05 was considered significant.

## Results

### Early results

The early mortality rate was 4.4% (4/90). They all belonged to the conventional group (*P* = .026) and died of low cardiac output syndrome (LCOS), which was related to early mortality (*P* < .001). CPB was used more frequently in the radical group, but CPB time and use of aortic cross-clamp were not different between groups. MELD (Model for End-stage Liver Disease) score was correlated with the amount of postoperative chest tube drainage. (*P* < 0.001, Correlation Coefficient = .470) However, the correlation between CPB time and the amount of postoperative chest tube drainage was not significantly correlated. (*P* = .222, Correlation Coefficient = .130). Also, postoperative CVP was not different between groups. Duration of stay in the intensive care unit was not different between groups, but hospital stay was longer in the conventional group. There was no intergroup difference in the number of early complications (Table 3), which correlated with the period of hospitalization (*P* = .002, ρ = .329).

**Table 3:** Operative Results

Variables	Conventional group	Radical group	*P* value
CPB, n (%)	26 (70.3%)	49 (92.5%)	.005^a^
CPB time, minutes	129.5 (90.0, 178.0)	119.0 (85.0, 177.0)	.644^b^
ACC, n (%)	4 (10.8%)	11 (20.8%)	.213^a^
ACC time, minutes	79 ± 14.8	68 ± 23.7	.407^b^
Concomitant operations	4 (10.8%)	16 (30.2%)	.025^a^
Postoperative CVP, mmHg	11.7 ± 5.3	10.8 ± 3.3	.381^b^
ICU stay, days	3.0 (2.0, 5.0)	2.0 (1.0, 3.0)	.086^c^
Hospital stay, days	26.5 (18.0, 49.0)	18.5 (11.0, 21.0)	.001^c^
The amount of postoperative transfusion			
*Red blood cell (unit)*	2 (0, 3.5)	2 (0, 4)	.824^c^
*Fresh frozen plasma (unit)*	0 (0, 3)	0 (0, 2)	.678 ^c^
*Platelet concentrate (unit)*	0 (0, 8)	0 (0, 2.5)	.309 ^c^
*Cryoprecipitate (unit)*	0 (0, 0)	0 (0, 0)	.840 ^c^
The amount of chest tube drainage (ml)	2040 (1100, 3815)	2400 (1490, 5597)	.367 ^c^
Numbers of early complications, n (%)	14 (37.8%)	12 (22.6%)	.118^a^
*Pericardial or pleural effusion*	2 (5.4%)	2 (3.8%)	1.000^d^
*Transient arrhythmia*	1 (2.7%)	4 (7.5%)	.645^d^
*Wound dehiscence*	1 (2.7%)	2 (3.8%)	1.000^d^
*Postoperative bleeding*	1 (2.7%)	1 (1.9%)	1.000^d^
*Pneumothorax*	1 (2.7%)	2 (3.8%)	1.000^d^
*Pneumonia*	0 (%)	2 (3.8%)	.510^d^
*Acute kidney injury*	2 (3.8%)	0 (%)	.166^d^
*Phrenic nerve palsy*	1 (2.7%)	0 (%)	.411^d^
*Mediastinitis*	1 (2.7%)	0 (%)	.411^d^
*Cerebrovascular accident*	1 (2.7%)	0 (%)	.411^d^
*Hepatic encephalopathy*	1 (2.7%)	0 (%)	.411^d^
*Low cardiac output syndrome, n (%)*	6 (16.2%)	3 (5.7%)	.153^d^

^a^Chi-squared test, ^b^Two-sample t-test, ^c^Mann-Whitney test, ^d^Fisher’s exact test

*ACC* aortic cross-clamp, *CPB* cardiopulmonary bypass, *CVP* central venous pressure, *ICU* intensive care unit

### Long-term outcomes

**Fig. 3:**
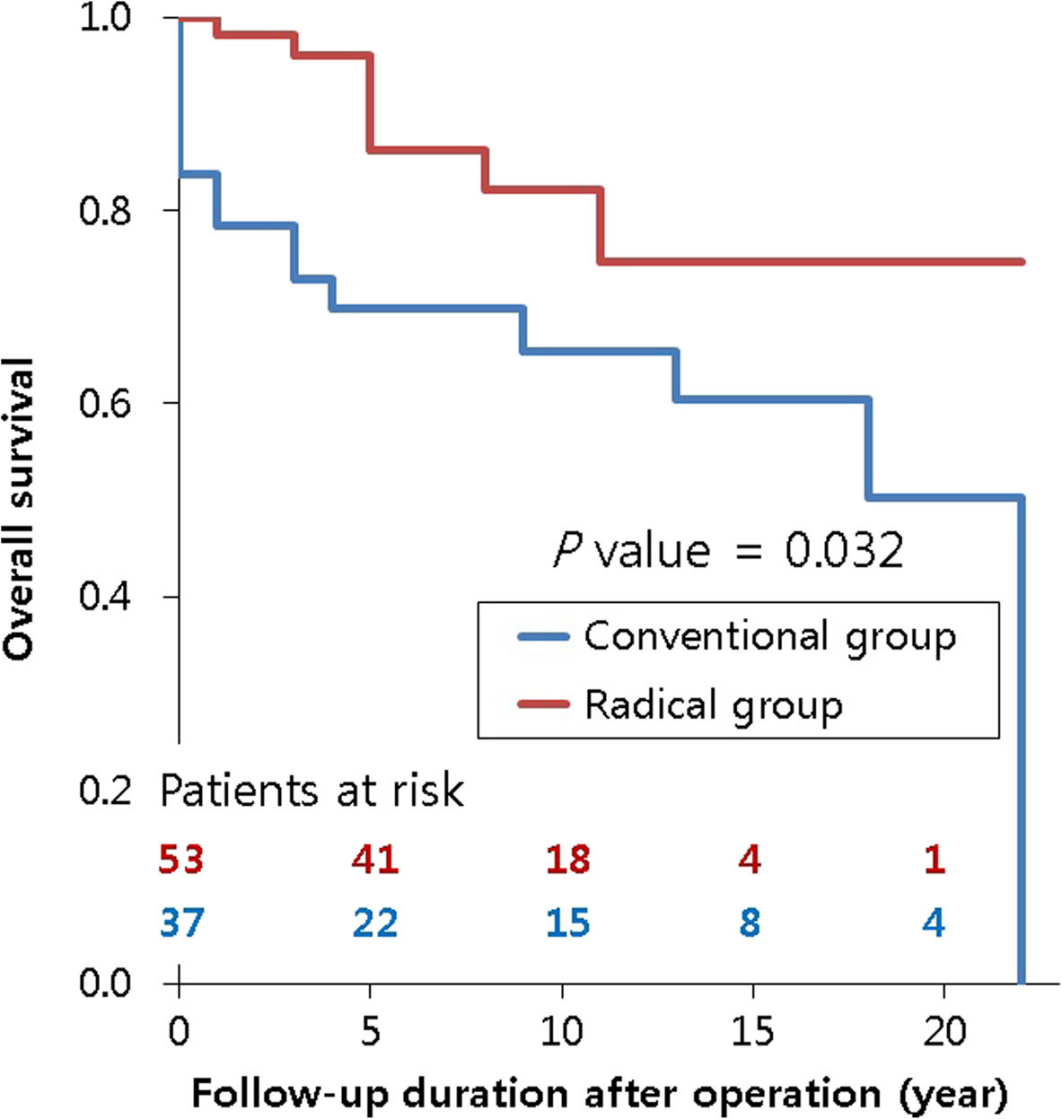
Survival rate of each group

**Fig. 4:**
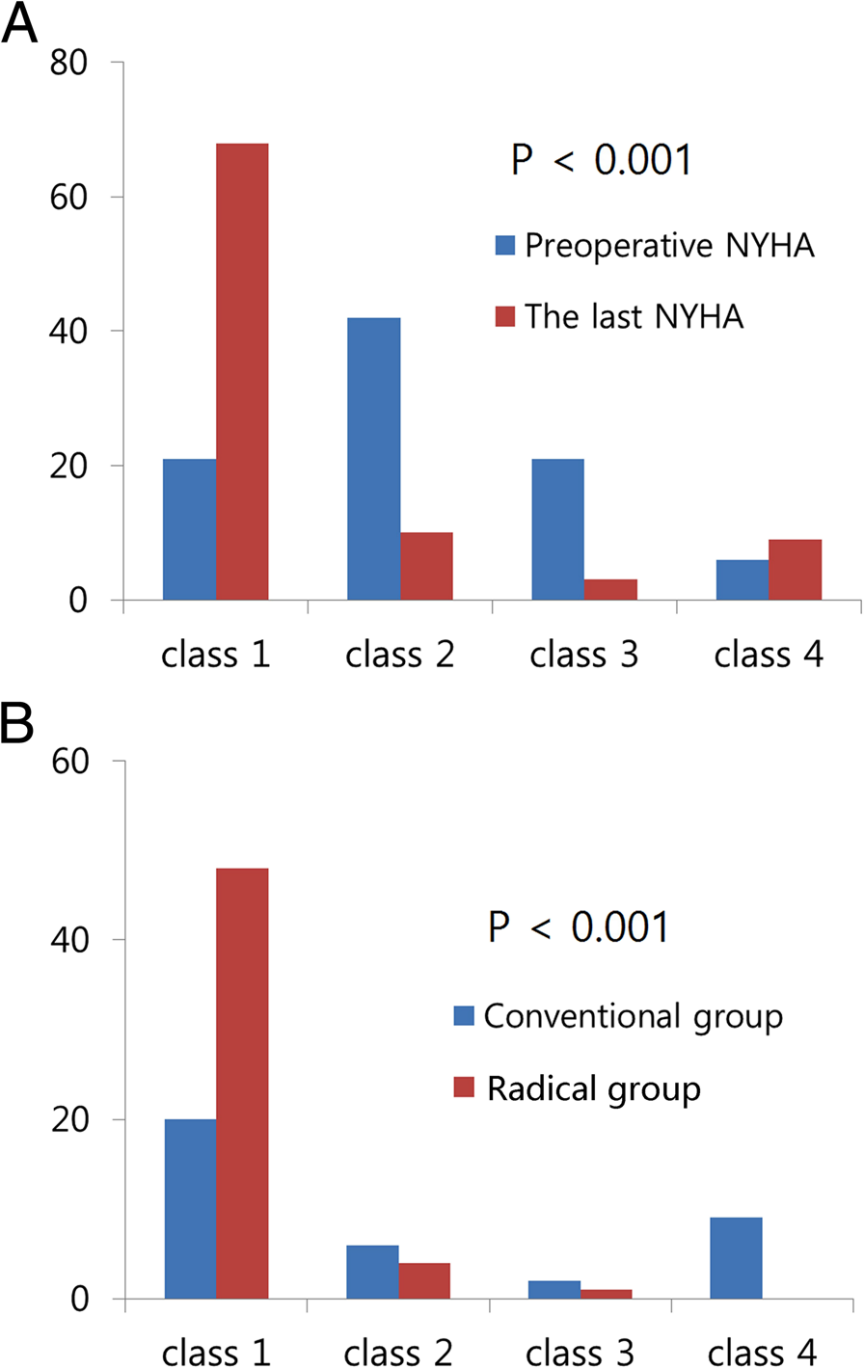
Functional recovery after pericardiectomy; (**a**) New York Heart Association (NYHA) class before and after the operation, (**b**) Final NYHA class according to the extent of pericardiectomy

The late mortalities occurred in 19 patients. The survival rate was higher in the radical group (*P* = .032, 50.4 ± 11.9% versus 74.7 ± 9.2% in 20 years, Fig. 3). NYHA classes of both groups had recovered until the last follow-up (*P* < .001), but the radical group showed better recovery (*P* < .001, Fig. 4). Conventional pericardiectomy (HR = 6.181; 95% CI (1.042, 36.656)), redosternotomy (HR = 6.441; 95% CI (1.224, 33.889) and preoperative grade of tricuspid regurgitation (TR, HR = 15.003; 95% CI (1.099, 204.894) were associated with late mortality (Table 4).

**Table 4:** Factors associated with long-term mortality. P values were calculated by the Cox proportional hazard model for multivariate analyses

	HR (95% CI)	*P* value
Age	1.074 (.981, 1.176)	.122
MELD (model for end-stage liver disease) score	1.095 (.852, 1.406)	.480
Preoperative NT-proBNP	1.000 (1.000, 1.000)	.463
Preoperative grade of tricuspid regurgitation	15.003 (1.099, 204.894)	.042
Redosternotomy	6.441 (1.224, 33.889)	.028
Conventional pericardiectomy	6.181 (1.042, 36.656)	.045
Postoperative central venous pressure	1.008 (.843, 1.206)	.927
The amount of the postoperative RBC transfusion	.946 (.731, 1.226)	.677
The amount of the postoperative PC transfusion	.937 (.838, 1.049)	.259
The amount of postoperative chest tube drainage	1.000 (1.000, 1.000)	.433
Postoperative low cardiac output syndrome	.763 (.077, 7.556)	.817

*CI* confidence interval, *HR* hazard ratio, *NT-proBNP* N-terminal prohormone of brain natriuretic peptide, *PC* platelet concentrate, *RBC* red blood cell

There were no echocardiographic parameters which improved in the conventional group only (Table 5). Right ventricular systolic pressure decreased, and pericardial thickening resolved only in the radical group with significant intergroup differences as time went on (Table 5). TR worsened after the operation in both groups, but it deteriorated more in the conventional group. TR grade improved over time in the radical group (Table 5).

**Table 5:** Summary of serial echocardiographic changes of each group (preoperative (Preop), postoperative (Postop) and the last (Last) echocardiographs). Plast mean P value after comparisons between the last variables of each group

Echocardiographic variables	Conventional group				Radical group				*P*last
	Preop	Postop	Last	*P*	Preop	Postop	Last	*P*	
LVEDD, mm	40 (37.3, 47.5)	44.2 ± 6.8	45.1 ± 4.9	.008^a^	43.5 ± 6.5	45.1 ± 5.8	48 (42.5,53)	.01^a^	.06^b^
TR 0	33 (89.2%)	13 (59.1%)	19 (59.4%)	.01^c^	52 (98.1%)	35 (79.5%)	44 (88.0%)	.03^c^	.004^c^
1	4 (10.8%)	8 (36.4%)	12 (37.5%)		1 (1.9%)	6 (13.6%)	5 (10.0%)		
2	0 (0%)	1 (4.5%)	1 (3.1%)		0 (0%)	3 (6.8%)	1 (2.0%)		
RVSP, mmHg	33.7 ± 9.5	30.6 ± 8.8	31 (26.9,37.3)	.75^a^	35.2 ± 8.1	31 (27,40)	26 (23,35.3)	.001^a^	.01^b^
Pericardial thickening	29 (80.6%)	13 (81.3%)	16 (76.2%)	.93^c^	49 (96.1%)	17 (73.9%)	17 (47.2%)	<.001^d^	.03^d^

*LVEDD* left ventricular end-diastolic diameter, *RVSP* right ventricular systolic pressure, *TR* tricuspid regurgitation, ^a^Kruskal-Wallis test, ^b^Mann-Whitney test, ^c^Fisher’s exact test, ^d^Chi-squared test

## Discussion

This study is one of only a few which compare long-term results between conventional and radical pericardiectomies. The main findings were as follows: (1) The survival rate was higher in the radical group. (2) Radical pericardiectomy was one of the factors associated with survival. (3) There was more improvement of certain echocardiographic parameters in the radical group as time passed.

Radical pericardiectomy was one of several factors associated with survival. Gongora et al. [12] previously showed pericardiectomy posterior to the phrenic nerves was a predictor of long-term survival. Chowdhury et al. [3] also confirmed conventional pericardiectomy is a risk factor for late mortality. The present study confirmed those previous reports. There are various kinds of surgical techniques of pericardiectomies according to the extent of pericardiectomy, surgical approach, or use of CPB. CPB is not used when thoracotomy is done [13, 14], but it is used when sternotomy is done [8, 15–17]. Although left thoracotomy enables pericardiectomy posterior to the left phrenic nerve without CPB, it is difficult to remove the pericardium around the right side of the heart [14, 18]. Chowdhury et al. [3] stated CPB is unnecessary even when total pericardiectomy is planned via a sternotomy, but total pericardiectomy by their definition is pericardiectomy anterior to the phrenic nerves. Therefore, in our opinion, sternotomy with CPB is inevitable if radical pericardiectomy is planned. Some authors have reported CPB is a risk factor for early mortality [5, 9], but the early mortality rate in our study was 1.3% (1/75) among the patients who underwent CPB. Bleeding risk is one of the complications of CPB especially if a patient has liver cirrhosis. In our results, the MELD score was correlated with the amount of postoperative chest tube drainage. (*P* < 0.001, Correlation Coefficient = .470) However, the correlation between CPB time and the amount of postoperative chest tube drainage was not significantly correlated. (*P* = .222, Correlation Coefficient = .130). On the other hand, there are several advantages of CPB. It helps to identify an appropriate dissection plane by emptying the ventricular cavities and helps to treat inadvertent cardiac injury [3, 9, 15, 16]. It also enables repair of the tricuspid valve without any hesitation [12]. Furthermore, Cho et al. [15] stated CPB can control preoperative volume overload.

Etiology, especially postradiative pericarditis has been known to be a predictor of late outcomes. Ling et al. [7] concluded radiation exposure was predictive of late mortality and other papers reported similar results [2, 4, 5, 12, 19, 20]. However, it was not found to be a factor associated with late mortality in our study. Patients who had postradiative pericarditis (5.6%) were too few to reach a meaningful conclusion [6].

Preoperative CVP has been known to be an important factor associated with survival. Elevated right atrial pressure and right ventricular end-diastolic pressure more than 20 mmHg were previously identified as risk factors for mortality [3, 8]. The preoperative CVP in our study was higher than normal range but under 20 mmHg. Either preoperative or immediate postoperative CVP was not a factor associated with survival. Preoperative CVP in the conventional group was higher than that in the radical group statistically (18.5 vs. 16.2 mmHg). One may think the mortality rate was higher in the conventional group because an elevated CVP indicates poor conditions; however, other clinical and echocardiographic findings were not different between groups, and a Cox proportional hazard model analyzes the data after removal of intergroup differences. We also wonder if the statistical difference between 18.5 and 16.2 mmHg is still significant in a clinical situation. The most (70%) preoperative CVP which were measured in the operating room under general anesthesia could be a limitation of our data because they can be influenced by mechanical ventilation, full sedation, or volume status at the moment.

The number of early complications was associated with later mortality in the univariate analysis but not in the multivariate analysis. It rather correlated with the period of hospitalization (*P* = .002, ρ = .329). Postoperative LCOS was more related with early mortality than late mortality. It was comparable to the previous reports in which postoperative LCOS affected early survival rather than late one [21, 22].

TR worsened after the operation in both groups, but it was aggravated more in the conventional group in the last echocardiography. Several cases of worsening TR or right ventricular dysfunction after pericardiectomy have been reported [23–25]. Mantri et al. concluded if an adequate pericardiectomy is done at an earlier stage, the regurgitations will regress [26]. Gongora et al. [12] stated TR is relieved in only 29% of patients after pericardiectomy, and they recommended repair of the tricuspid valve because TR of more than a moderate grade increases late mortality. We experienced a case of tricuspid valve repair because TR was aggravated six years after pericardiectomy alone. Therefore, we expect postoperative TR can be prevented if a surgeon repairs the valve more actively. The preoperative grade of TR was also verified as a factor associated with late mortality in our study. Nevertheless we should be careful not to interpret it to mean tricuspid valve repair would improve late survival. However we may make a hypothesis that postoperative aggravation of TR could be a sign of another pathology which was masked by constrictive pericarditis or made by inadequate pericardiectomy. Then the aggravated TR after pericardiectomy in our study could be explained if the hypothesis is demonstrated someday.

There were some limitations to this study. First, the small number of patients and retrospective study design did not allow us to draw any definite conclusions. The survival rate of radiation-induced constrictive pericarditis is known to be low [20] but it was not in this study. We think the ratio of radiation-induced pericarditis was too low in Korean patient population. The other Korean authors also reported such unique Korean environment could be a limitation in their article [6]. Second, since a multivariate analysis is most robust when it involves at least 10 events for each variable assessed, it seems likely our multivariate analyses were over-fitted. So a larger number of patients and a larger number of adverse outcomes are needed to perform these analyses later. Third, clinical implication of this study remains unclear yet although the findings of this study were reasonable. So prospective randomized data are necessary to establish the clinical implication.

## Conclusions

Radical pericardiectomy led to greater improvement in right ventricular systolic pressure and lesser deterioration of TR with the passage of time than did the conventional procedure. Conventional pericardiectomy and preoperative higher grade TR were associated with long-term mortality.

## Additional files

Additional file 1: The number of each operative technique which was performed every year. (JPG 64 kb)

Additional file 2: Factors for the univariate analyses and their *P* values after the analyses. (DOCX 16 kb)

## Abbreviations

CPB: Cardiopulmonary bypass
CVP: Central venous pressure
LCOS: Low cardiac output syndrome
NYHA: New York Heart Association
