# Supplementary figures and images for: Long-term results of radical pericardiectomy for constrictive pericarditis in Korean population
Source: J Cardiothorac Surg. 2019 Feb 6;14:32. doi: 10.1186/s13019-019-0845-7 (PMC6364466; doi:10.1186/s13019-019-0845-7)

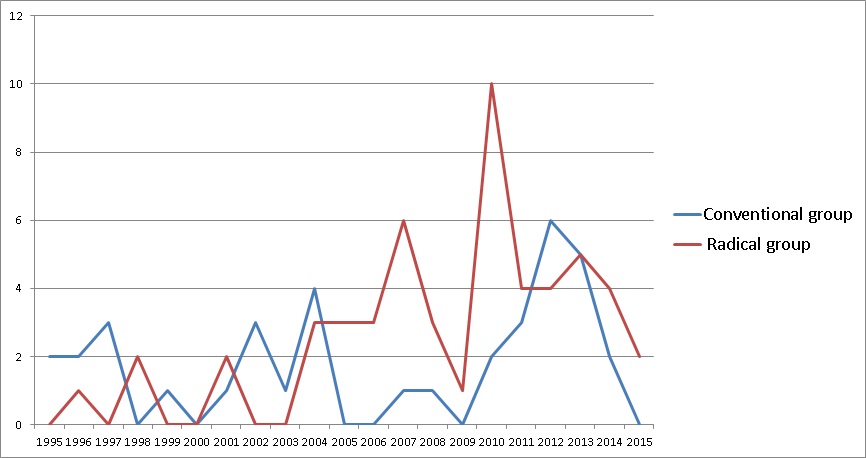

Supplement: Supplementary file 1 — The number of each operative technique which was performed every year. (JPG 64 kb) [file 13019_2019_845_MOESM1_ESM.jpg]
