# Supplementary material for: Long-term results of radical pericardiectomy for constrictive pericarditis in Korean population
Source: J Cardiothorac Surg. 2019 Feb 6;14:32. doi: 10.1186/s13019-019-0845-7 (PMC6364466; doi:10.1186/s13019-019-0845-7)
Supplement: Supplementary file 2 — Factors for the univariate analyses and their P values after the analyses. (DOCX 16 kb) [file 13019_2019_845_MOESM2_ESM.docx]

Supplementary Material 2 Factors for the univaritae analyses and their P values after the analyses

|  | Continuous variables | | Categorial variables | |
| --- | --- | --- | --- | --- |
| Preoperative findings | Age | .027 | Sex | .237 |
|  | ICU stay | .001 | Diabetes mellitus | .805 |
|  | Serum Hemoglobin |  | Hypertension | .831 |
|  | Serum sodium | .002 | Cerebrovascular accident | <.001 |
|  | Serum albumin |  | Coronary artery disease | .601 |
|  | Serum bilirubin | .481 | Chronic renal failure on dialysis | .586 |
|  | Serum creatitine | .042 | Liver cirrhosis | .387 |
|  | Serum NT-proBNP | .078 | Ascites | .604 |
|  | MELD score | .029 | Atrial fibrillation | .762 |
|  | CVP | .482 | Previous open heart surgery | <.001 |
|  |  |  | NYHA class | .935 |
|  |  |  | Idiopathic pericarditis | .530 |
|  |  |  | Tubercuolous pericarditis | .0038 |
|  |  |  | Malignant pericarditis | .002 |
|  |  |  | Radiation-induced pericarditis | .638 |
| Preoperative echocardiographic parameters | LVEF | .453 | Grade of TR | .005 |
|  | RVSP | .839 | Grade of MR | .491 |
|  |  |  | Pericardial thickening | .682 |
|  |  |  | Diastolic flattening of LV posterior wall | .112 |
|  |  |  | Ventricular septal wall motion abnormality | .315 |
| Operative findings | CPB time | .373 | Redo-sternotomy | .001 |
|  | ACC time | .620 | Conventional pericardiectomy | .229 |
|  |  |  | The use of CPB | .812 |
|  |  |  | The usde of ACC | .428 |
| Postoperative findings | CVP | .144 | Low cardiac output syndrome | .119 |
|  | ICU care days | .001 | Early complications | .135 |
|  | Total hospital days | <.001 |  |  |
|  | RBC transfusion | .198 |  |  |
|  | FFP transfusion | .900 |  |  |
|  | PC transfusion | .091 |  |  |
|  | Cryo transfusion | .018 |  |  |
|  | Chest tube drainage | .043 |  |  |

ACC, aortic cross-clamp; CPB, cardiopulmonary bypass; Cryo, cryoprecipitate; CVP, central venous pressure; FFP, fresh frozen plasma; ICU, intensive care unit; IVC, inferior vena cava; LV, left ventricular; LVEF, left ventricular ejection fraction; MELD, model for end-stage liver disease; MR, mitral regurgitation; NT-proBNP, N-terminal prohormone of brain natriuretic peptide; NYHA, New York Heart Association; PC, platelet concentrate; RBC, red blood cell; RVSP, right ventricular systolic pressure; TR, tricuspid regurgitation.
